# Catheter ablation for atrial tachycardia in pediatric patients: a single-center experience

**DOI:** 10.3389/fcvm.2024.1436241

**Published:** 2024-11-20

**Authors:** Ruoyu Chen, Xin Xu, Shuang He, Qian Liu, Lin Liu, Qin Zhang, Tiewei Lu

**Affiliations:** ^1^ Department of Cardiology, Children’s Hospital of Chongqing Medical University Chongqing, China; ^2^ Ministry of Education Key Laboratory of Child Development and Disorders, China International Science and Technology Cooperation Base of Child Development and Critical Disorders, Chongqing Key Laboratory of Pediatrics, Chongqing, China

**Keywords:** pediatrics, atrial tachycardias, radiofrequency catheter ablation, tachycardia-induced cardiomyopathy, antiarrhythmic drugs

## Abstract

**Purpose:**

Atrial tachycardia is an uncommon supraventricular tachycardia in children. It is often drug-resistant and likely to occur concomitantly with tachycardia-induced cardiomyopathy, making radiofrequency catheter ablation the preferred treatment. The aim of this study was to assess the feasibility, safety, and effectiveness of radiofrequency catheter ablation for the treatment of different types of atrial tachycardia in children, particularly in those with drug-resistant and tachycardia-induced cardiomyopathy.

**Methods:**

A total of 28 children with atrial tachycardia (including focal atrial tachycardia and atrial flutter) who underwent atrial radiofrequency ablation at the Children's Hospital Affiliated to Chongqing Medical University from May 2018 to December 2023 were included. The baseline characteristics, preoperative medication, surgical information, and postoperative follow-up data of these children were analyzed statistically.

**Results:**

The mean age patients at ablation was 10.24 ± 3.40 years. A total of 78.6% of the patients (22/28) who received preoperative pharmacological treatment had intermittent or persistent atrial tachycardia. Of the 28 children who underwent radiofrequency ablation, 24 (85.7%) were diagnosed with focal atrial tachycardia, three (10.7%) with atrial flutter, and one (3.6%) with both. No postoperative complications occurred in any patient. The immediate ablation success rate in the 25 patients with focal atrial tachycardia was 96.0% (24/25). After 26.89 ± 18.17 months of follow-up, only three patients had recurrence. The ablation difficulty of focal atrial tachycardia originating in the appendage was higher than that originating in the non-atrial appendage (44.4% vs. 6.3%, *p* = 0.01). The success rate of ablation for atrial flutter was 100%, except in one child with underlying cardiomyopathy who experienced recurrence. Final success was achieved in 25 of the 28 patients (89.2%) at the end of the follow-up period. In addition, eight children (28.6%) in this study were diagnosed with tachycardia-induced cardiomyopathy, with significantly increased ejection fraction and shortening rate after radiofrequency ablation (*p* < 0.01), whereas the left ventricular end-systolic diameter were not significantly reduced during the follow-up period (*p* > 0.05).

**Conclusion:**

Radiofrequency catheter ablation is safe and effective for the treatment of atrial tachycardia in children in the short- and long-term.It can be used as the first treatment option for children with medically refractory atrial tachycardia and tachycardia-induced cardiomyopathy.

## 1 Introduction

Atrial tachycardia (AT) is uncommon in children, accounting for approximately 10%–15% of all supraventricular tachycardias (SVTs) (1). According to current studies, while ATs in most infants can spontaneously resolve or be medically controlled, ATs in children older than three years are often more refractory and tend to be drug-resistant (2, 3). In addition, due to their insidious onset and limited capacity for expression, approximately 28%–30% of children with ATs will finally develop tachycardia-induced cardiomyopathy (TIC) (4, 5). In terms of treatment, radiofrequency catheter ablation (RFCA) has become the first-line treatment for SVTs in older children (age ≥3 years, weight ≥15 kg), and recent studies have shown that the application of three-dimensional mapping systems provides more assurance of the effectiveness of RFCA whilst reducing the risk of radiation exposure (6, 7). However, reports on the use of RFCA for pediatric patients with AT remain limited.

This study included children with focal atrial tachycardia (FAT) and atrial flutter (AFL) who underwent RFCA at a single center. We collected and analyzed their baseline characteristics, pre-procedure medication history, ablation information, and post-procedure follow-up data to investigate the effectiveness and safety of RFCA for pediatric AT, especially for those who are drug-resistant and have TIC.

## 2 Methods

### 2.1 Study population

This single-center retrospective study evaluated 28 children who underwent RFCA for FAT and AFL using a three-dimensional mapping system Carto3 (Biosense Webster Inc., Diamond Bar, CA, USA) at the Children's Hospital of Chongqing Medical University between May 2018 and December 2023. Children who underwent ablation for the first and second time were evaluated separately to avoid bias. The study was approved by the Clinical Ethics Committee of Children's Hospital of Chongqing Medical University, and informed consent was obtained from the guardians of all children.

The diagnoses of FAT and AFL were confirmed by at least two electrophysiologists based on the presence of electrocardiography (ECG), Holter, or electrophysiological examination results consistent with the diagnostic criteria (8, 9). TIC was defined by: (1) uncontrolled AT; (2) left ventricular dysfunction, including a left ventricular ejection fraction (LVEF) <50% and a short-axis shortening rate (FS) <28%, with or without dilation of the heart chambers; (3) improvement in left ventricular systolic function (LVEF ≥55% and FS ≥28%) after control of AT (4, 5). Drug refraction was defined as the presence of incessant or frequent episodes of AT after at least 1 month of standardized use of antiarrhythmic drugs (AADs) or AT relapse after successful drug control.

The inclusion criteria were: (1) 3–18 years of age; (2) AT confirmed by 12-lead surface ECG, Holter test, or intracardiac electrophysiological examination; (3) uncontrolled AT prior to RFCA. The exclusion criteria were: (1) no spontaneous or inducible AT observed during RFCA; (2) presence of other diseases that significantly affect cardiac structure or function, such as severe cardiac disease and other systemic diseases.

### 2.2 Electrophysiological examination and RFCA

AADs were discontinued for at least five half-lives. The Carto3 system (Biosense Webster Inc.) was used in all patients who underwent cardiac electrophysiology examination and RFCA according to standard operating procedures (6), and coronary venography was routinely performed to clarify whether the coronary veins were abnormal. For the left-sided foci, an atrial septal puncture into the left atrium was performed through the patent foramen ovale or under radiographic guidance. Appropriate bilateral femoral vein sheaths were selected according to the age and weight of each patient, through which a decapolar electrode, His electrode, and quadripolar electrodes were placed into the coronary sinus, right atrium, and right ventricle to simultaneously obtain surface and endocardial electrocardiograms.

Electrophysiological examinations were also performed. Through the right femoral vein vascular sheath, the diagnostic/ablation steerable catheter was gently moved up into the right atrium using anteroposterior and lateral views, and the anatomical structures of the superior vena cava, right atrium, and tricuspid annulus were constructed. His signals were marked on the right atrium, and atrial excitation was marked during tachycardia episodes to clarify the sequence of atrial excitation and determine the mechanism of tachycardia onset. If adequate atrial activity was not spontaneously present at the beginning of the procedure, standard atrial and ventricular pacing maneuvers were performed to induce AT and to exclude the possibility of other supraventricular tachycardias, and isoprenaline was used to stimulate atrial ectopy when necessary. There were also some cases in our practice in which AT was not induced. AT in children mostly occurs because of an abnormal, nonsinus, atrial focus with enhanced automaticity. According to previous studies (10, 11), the automaticity has been proven to be suppressed by anesthesia strategy, depth of anesthesia, and anesthetic agents occasionally, leading to a negative electrophysiological study for approximately 20% of ATs. In our study, we would terminate our ablation to avoid further injury when we found the inducibility of AT impossible during RFCA.

#### 2.2.1 Focal atrial tachycardia

Point-by-point high-density mapping was performed in the areas with early activation. The locations of the potential targets were identified by measuring the activation time, which was at least 30 ms ahead of the onset of the stable P wave. Additionally, a pure negative deflection (QS pattern) on a unipolar atrial electrogram was also detected. Color-coded three-dimensional mapping was used for identifying potential target sites. The selection of pressure or non-pressure sensing ablation catheter was based on the property of the ectopic foci, with radiofrequency energy delivered at a target temperature of 40℃ and a power of 25–35 W for a maximum of 60 s with a preset saline flow of 17 ml/min. The entire process was conducted under continuous temperature and impedance-monitoring conditions. If the tachycardia did not terminate within 10 s, the target point was re-mapped. After termination of tachycardia, multipoint ablation was performed around the target point. The patients were observed for 30 min after ablation. Finally, the endpoint of ablation was achieved when no tachycardia occurred after repetitive standard atrial and ventricular pacing maneuvers and the use of isoprenaline. If tachycardia recurred, the mapping and ablation procedures were repeated (12).

#### 2.2.2 Atrial flutter

RFCA for AFL was performed according to the standard protocols to delineate the tachycardia circuit and identify the slow conduction zone. Concealed entrainment from the tricuspid isthmus was performed to confirm or exclude the cavotricuspid isthmus (CTI) as the protected slow conduction zone. A point with a post-pacing interval minus a tachycardia cycle circumference of less than 30 ms was considered part of the refractory circuit. For CTI-dependent AFL, linear ablation was performed in the CTI at 30–35 W with power-controlled mode, and the temperature and saline irrigation speed was set at 40°C and 17 ml/min respectively for a maximum of 60 s per lesion until tachycardia was terminated and a bidirectional conduction block (≥130 ms) from the tricuspid annulus to the inferior vena cava was established. In patients without CTI-dependent AFL, radiofrequency energy was delivered to the protected and narrowest part of the slow-conduction zone, including the myocardial scar and tricuspid annulus, inferior vena cava, or superior vena cava. The ablation was terminated when a complete bidirectional block along these lines was achieved. The post-ablation study was performed 30 min later. In accordance with the standard protocols, successful ablation was determined after confirmation of flutter termination and persistent bidirectional block as assessed by pacing maneuvers (9, 13).

### 2.3 Complications and follow-up

Complications were defined as events directly related to RFCA that required further intervention, prolonged hospitalization, and/or could negatively affect long-term follow-up, and these included type II and/or III AV block, perforation, pericardial effusion, thrombus formation, damage to the phrenic nerve, stroke, and cardiac arrest (12).

All children underwent transthoracic and 12-lead ECG examination 1–2 days after RFCA, and Holter monitoring was reviewed if the results were suspicious. The same examinations were repeated at the follow-up visits at 1, 3, 6, and 12 months after the procedure, and then every 6 to 12 months after that. When children were not followed-up at scheduled intervals, guardians were contacted via telephone determine whether any related complications or recurrences had occurred. During follow-up, recurrence was defined as the reappearance of documented AT on ECG or Holter examination after successful ablation.

### 2.4 Statistical analyses

Continuous data of normal distribution are expressed as mean ± standard deviation (SD). Categorical data are expressed as quantities (percentages). Baseline comparisons of numerical variables were performed using an unpaired Student's *t*-test, while multiple groups of data were compared using one-way analysis of variance (ANOVA). Categorical variables are presented as counts (percentages) and were analyzed using Fisher's exact test. Statistical significance was set at *p* < 0.05. Statistical analyses were performed using the SPSS software (version 27.0; SPSS, Inc., Chicago, IL, USA).

## 3 Results

### 3.1 Patient characteristics

The baseline patient characteristics are shown in Table 1. The study included 28 children (12 male and 16 female; mean weight 34.98 ± 14.94 kg). The mean age at diagnosis was 8.94 ± 3.66 years, and the mean age at the time of procedure was 10.24 ± 3.40 years. Among the ATs diagnosed, 85.7% (24/28) were FAT, 10.7% (3/28) were AFL, and 3.6% (1/28) were a combination of FAT and AFL. All children presented with various clinical symptoms before consultation, mostly left ventricular systolic dysfunction (palpitations and chest tightness, 78.6%), with or without heart failure symptoms (fatigue and reduced activity tolerance, 53.6%). Another 10 children (35.7%) had other symptoms such as dizziness, headache, nausea, and vomiting. Through pre-procedure examinations, most patients (20 cases, 71.4%) were diagnosed by ECG alone, incessant tachycardia was monitored in 13 patients (46.4%), and TIC was diagnosed in eight patients at the initial diagnosis (28.6%). No severe underlying cardiac pathology was observed, and simple congenital heart disease was present in three patients (10.7%). The history of myocarditis, cardiac manipulation (surgical procedures and radiofrequency ablations), and severe events are described below.

**Table 1:** Baseline characteristics.

Characteristics	*n* = 28
Male	12 (42.9%)
Age at onset (years)	8.94 ± 3.66
Age at ablation (years)	10.24 ± 3.40
Weight (kg)	34.98 ± 14.94
Symptoms	
Left ventricular systolic dysfunction	23 (82.1%)
Heart failure symptoms	15 (53.6%)
Other symptoms	10 (35.7%)
Diagnostic examination	
ECG	20 (71.4%)
ECG + Holter	5 (17.9%)
Holter	2 (7.1%)
Electrophysiological Study	1 (3.6%)
Incessant ATs	13 (46.4%)
TIC	8 (28.6%)
Percentage of ATs	
FAT	24 (85.7%)
AFL	3 (10.7%)
FAT + AFL	1 (3.6%)
Past history	
Simple heart defects	3 (10.7%)
Myocarditis	7 (25.0%)
Previous heart operation	4 (14.3%)
Severe events	6 (21.4%)

Values are presented as *n* (%) or mean ± SD.

ECG, electrocardiography; AT, atrial tachycardia; TIC, tachycardia-induced cardiomyopathy; FAT, focal atrial tachycardia; AFL, atrial flutter.

Other symptoms include dizziness and headache, nausea and vomiting, abdominal pain, and syncope; incessant atrial tachycardia: atrial rhythms account for >90% of all cardiac beats throughout the day; simple heart defects: congenital cardiac defects that do not affect atrioventricular connections, position of the atrioventricular valve and venous reflux, history of previous cardiac manipulation including a history of radiofrequency ablation, interventional closure, and surgical intervention; and severe events, including cardiogenic shock and use of ventricular support related to AT.

### 3.2 Use of AADs

Except for four children at initial diagnosis and two who refused medical treatment, the remaining 22 children (78.6%) received single or combined AADs for at least 1 month before RFCA. After at least 1 month of drug treatment, only partial atrial control was achieved in 10 patients (35.7%). Moreover, two of eight patients with TICs (25.0%) had improved left heart systolic function after medical therapy, and the RFCA was performed for tachycardia that were still present.

### 3.3 Radiofrequency catheter ablation

#### 3.3.1 Focal atrial tachycardia

**Figure 1:**
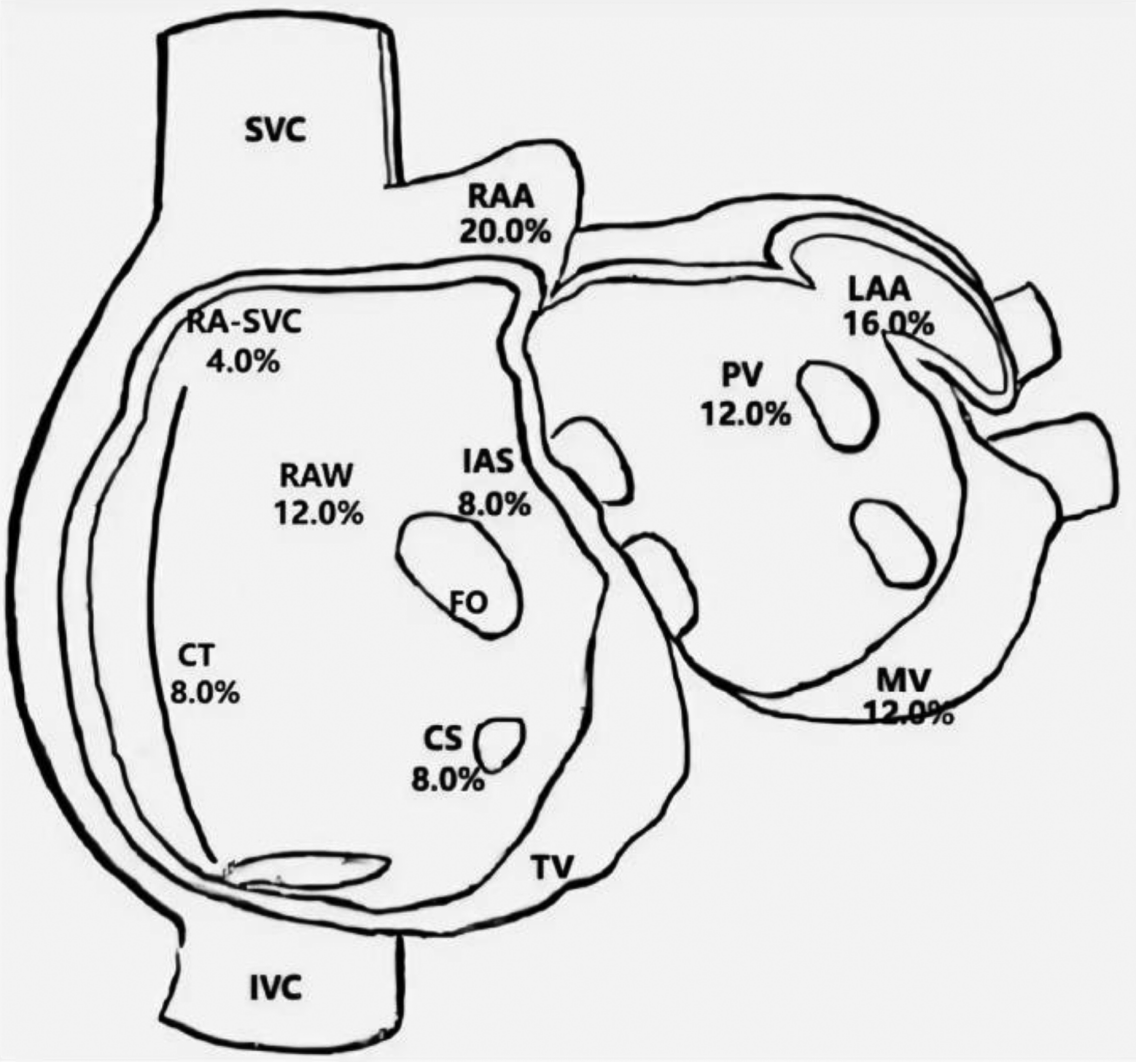
Distribution of atrial foci. CS, coronary sinus ostium; CT, crista terminalis; FO, foramen ovale; IAS, interatrial septum; IVC, inferior caval vein; LA, left atrium; LAA, left atrial appendage; MV, mitral valve; PV, pulmonary veins; RA-SVC, junction of right atrium and superior caval vein; RAA, right atrial appendage; RAW, right atrial wall; SVC, superior caval vein; TV, tricuspid valve.

Twenty-five patients with clinically-documented FAT were included in this study, 24 of whom were diagnosed preoperatively and one who was diagnosed during electrophysiological examination. Spontaneous tachycardia was present in 11 of the 25 patients (44.0%) in the electrophysiological study, while tachycardia was induced by stimulation in the remaining patients. The distribution of atrial foci identified by three-dimensional mapping is shown in Figure 1. The atrial appendage accounted for the largest proportion (20.0% in the right appendage and 16.0% in the left appendage), while other locations included the right atrial wall (free and posterior walls) (12.0%), mitral annulus (12.0%), pulmonary veins (12.0%), crista terminalis (8.0%), coronary sinus (8.0%), and interatrial septum (8.0%).

The patients with FAT were divided into an atrial appendage tachycardia (AAT) group (*n* = 9, 36.0%) and a non-AAT group (*n* = 16, 64.0%), as shown in Table 2. No difference was observed between the two groups in terms of sex composition, percentage of early FAT onset (age of onset <7 years) or the number of patients with incessant AT (66.7% vs. 31.3%, *p* = 0.115). According to procedural information, there was no significant difference in the percentage of foci of both atria between the two groups (55.6% vs. 56.3%, *p* = 1.000) or procedure time (235.00 ± 96.70 min vs. 246.88 ± 64.98 min, *p* = 0.716). During the procedure, except for one child in the AAT group who experienced failed ablation, the acute success rates between the two groups were not significantly different (88.9% vs. 100%; *p* = 0.120). However, over a mean follow-up of 24.44 ± 19.98 and 23.19 ± 11.50 months, respectively in the AAT and non-AAT groups, three children with AAT experienced recurrences within 3 months after the procedure. These failed operations and relapse cases suggest that the operations were obviously more difficult in patients with AAT than in those without AAT (44.4% vs. 0%, *p* = 0.01).

**Table 2:** Comparison of AAT and non-AAT (*N* = 25).

	AAT (*n* = 9)	non-AAT (*n* = 16)	*p* value
Male (%)	5 (55.6%)	6 (37.5%)	0.434
Onset <7 years (%)	4 (44.4%)	6 (37.5%)	1.000
Incessant FAT (%)	6 (66.7%)	5 (31.3%)	0.115
TIC (%)	2 (22.2%)	5 (31.3%)	1.000
Ectopic foci (RA) (%)	5 (55.6%)	9 (56.3%)	1.000
Procedure time (min)	235.00 ± 96.70	246.88 ± 64.98	0.716
Acute success (%)	8 (88.9%)	16 (100.0%)	0.120
Follow-up (months)	24.44 ± 19.98	23.19 ± 11.50	0.842
Failed operations and recurrences (%)	4 (44.4%)	0	0.01

Values are presented as the mean ± SD or *n* (%).

AA, atrial appendage; AAT, atrial appendage tachycardia; TIC, tachycardia-induced cardiomyopathy.

Two patients with relapse underwent successful secondary radiofrequency ablation without recurrence during the 4-month and 24-month follow-up, resepectively. Two other cases of failed ablation and recurrence showed infrequent FAT episodes without any clinical manifestations or hemodynamic changes; therefore, they were treated with AADs based on comprehensive consideration by electrophysiologists and guardians. These two patients responded well to postoperative pharmacological treatment. A reduction in arrhythmia burden and improvement in TIC was observed with the use of sotalol in one patient, and rhythm and heart rate were controlled satisfactorily with combined use of metoprolol with propafenone in the other patient.

#### 3.3.2 Atrial flutter

**Figure 2:**
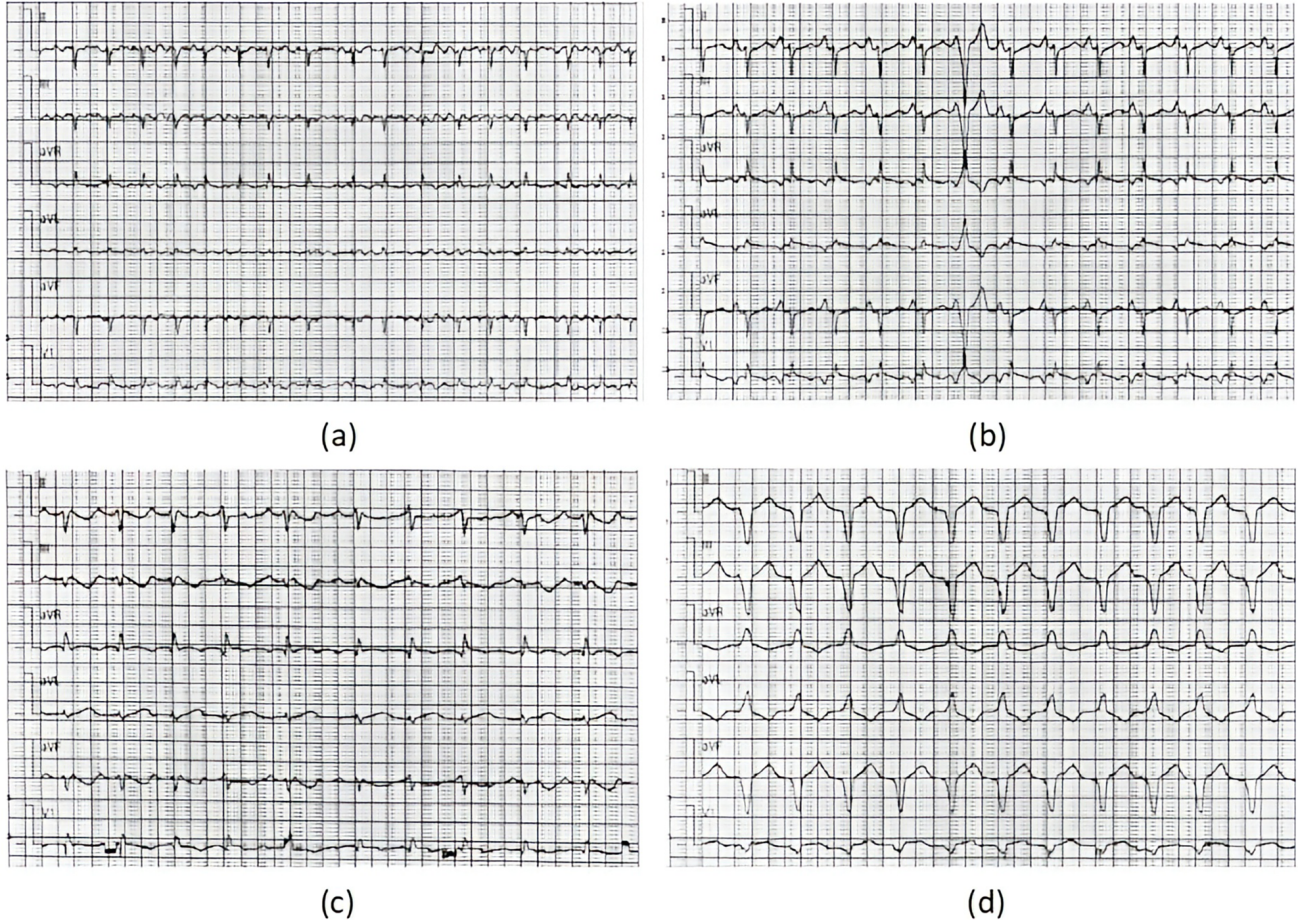
ECG results of the ARCM patient. ECG results: **(a)** moments before the procedure (AFL), **(b)** immediately after RFCA (1 day postoperatively, sinus tachycardia) **(c)** 1 year after RFCA (recurrence of AFL), **(d)** at the recent follow-up of the study (persistent ventricular tachycardia).

**Figure 3:**
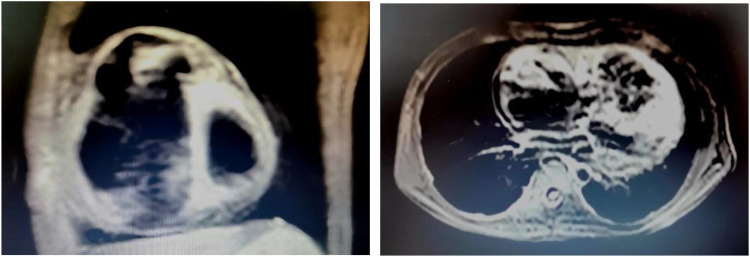
MRI results of the ARCM patient. MRI examination was performed 1 year after RFCA, at the time the recurrence of atrial flutter was detected in this child. At the same time, there was also an abnormal enlargement of the right ventricle and the presence of paroxysmal ventricular tachycardia. The result showed that the right atrium and right ventricle were significantly enlarged, the right ventricular wall was thinned, no exact abnormal fat signal was seen, and the right ventricular trabeculae were increased and thickened.

A total of four patients (two male and two female; mean age at diagnosis 11.90 ± 1.66 years) with AFL were included. Among them, one patient had a history of cardiac surgery, one had a history of fulminant myocarditis and patent foramen ovale, and the other was diagnosed with cardiomyopathy during follow-up. RFCA was performed during the first visit in half of the patients, and the other two patients received medical treatment for 3 and 24 months, respectively. AFL was incessant in three patients, one of whom had coexisting TIC. Four patients with AFL were included in this study, and all of them were found to have CTI-dependent AFL on electrophysiological examination, including one who had an AFL circuit formed around the right atrial surgical incision. The mean procedure time was 153.75 ± 68.11 min, and all procedures were successful. During a follow-up of more than 2 years, only the patient with a combination of FAT and AFL experienced recurrence, accompanied by an abnormal enlargement of the right ventricle and the presence of paroxysmal ventricular tachycardia despite regular treated with AADs. Eventually, the child was diagnosed with arrhythmogenic cardiomyopathy, which may be highly correlated with recurrence (14) (Relevant ECG and MRI results shown in the Figures 2, 3).

### 3.4 Complications and postoperative follow-up

Acute results and follow-up of ablation (including the first and second ablations) were collected from all patients. No significant complications were observed in any patient. Moreover, an immediate procedural success rate of 96.7% (29/30) and a final success rate of 89.2% (25/28) were achieved during long-term follow-up.

**Figure 4:**
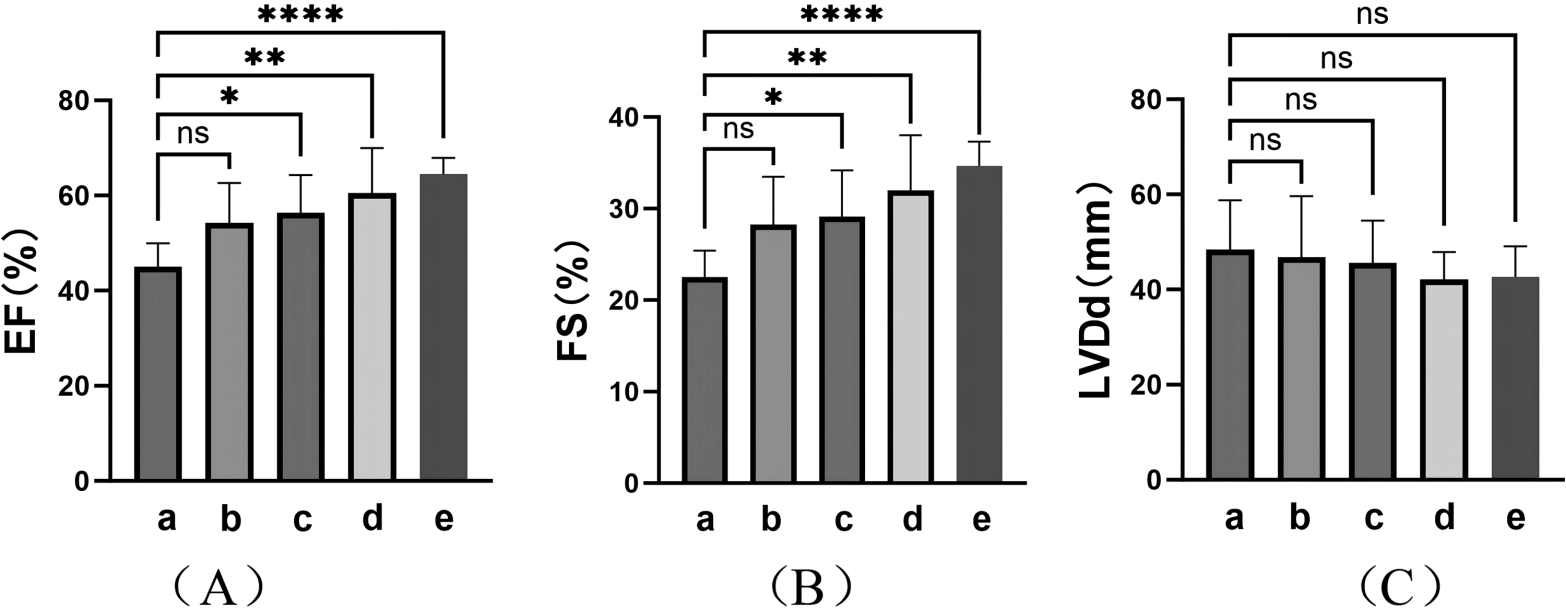
Cardiac ultrasound findings in TIC patients. Indicators of left heart function: **(A)** Ejection fraction (EF); **(B)** Fraction shorting (FS); **(C)** Left ventricular diastole diameter (LVDd). (a–e) represent the results of cardiac ultrasound at the time of diagnosis of TIC (8, 100%), moments before procedure (8, 100%), immediate moments after RFCA (1–2 days postoperatively) (8, 100%), short-term moments after RFCA (1–3 months post-procedure) (6, 75.0%), and final follow-up of the study (>3 months post-procedure) (6, 75.0%) respectively. ****: *p* < 0.001; **: *p* < 0.01; *: *p* < 0.05; ns, no significance.

TIC was observed in eight patients (28.6%) at the initial visit. Analysis was performed on the cardiac ultrasound results of these patients at the time of TIC diagnosis, before the procedure, 1–2 days after RFCA, 1–3 months after RFCA, and at the final follow-up of the study (Figure 4 and Table 3). The results showed a significant improvement in the left heart systolic function after treatment (Figures 4A,B; F_(EF)_ = 7.385, F_(FS)_ = 7.065, *p* < 0.01). A significant increase in left ventricular systolic function was observed in these patients (*p* < 0.01), but no significant decrease in left ventricular end-systolic diameter (LVDd) was observed (*p* > 0.05). In detail, all patients received at least 1 month of treatment with AADs over an average period of 23.63 ± 21.17 months before ablation with unsatisfactory efficacy. In contrast, RFCA was a more efficacious treatment for significant EF and FS improvements. Except for two patients, one who showed improvement in TIC with AAD treatment only and one who was not followed up regularly, cardiac function returned to normal level at 3.80 ± 2.77 months after RFCA in the remaining patients (five of eight). Further improvements in cardiac function were observed during the postoperative follow-ups, with more prominent results between the preoperative period and the last follow-up (*p* < 0.001). However, no significant improvement in LVDd was observed during the course of treatment in these patients, despite a numerical decrease [Figure 4C; F_(LVDd)_ = 0.527, *p* > 0.05].

**Table 3:** Cardiac ultrasound findings in patients with TIC.

	a	b	c	d	e	F value	*p* value
EF (%)	45.00 ± 4.99	54.25 ± 8.38	56.38 ± 7.95	60.50 ± 9.46	64.50 ± 3.39	7.385	<0.01
FS (%)	22.50 ± 2.93	28.25 ± 5.23	29.13 ± 5.06	32.00 ± 6.00	34.67 ± 2.66	7.065	<0.01
LVDd (mm)	48.38 ± 10.42	45.63 ± 13.05	45.63 ± 8.86	42.17 ± 5.74	42.83 ± 6.71	0.527	0.72

Values are presented as the mean ± SD.

Cardiac ultrasound results: (a) at the time of TIC diagnosis (8, 100%), (b) moments before the procedure (8, 100%), (c) immediately after RFCA (1–2 days postoperatively) (8, 100%), (d) more than 1 month after RFCA (1–3 months post-procedure) (6, 75.0%), and (e) at the final follow-up of the study (>3 months post-procedure) (6, 75.0%).

## 4 Discussion

### 4.1 Drug refractoriness of ATs

AT, which accounts for approximately 10%–15% of all SVTs, are relatively uncommon in children (1), and medical therapy is often used as the first choice of treatment for these patients. According to the JCS/JHRS guideline recommendations and the findings of relatively long-term studies (3, 13, 15–17), class Ic and class II AADs are recommended as the first choice for pediatric FAT, either alone or in combination. Class III drugs are only recommended for patients with poor ventricular function for short-term use or as second-line drugs because of their serious side effects. Medicines for AFL should be carefully selected on an individual basis. Considering that children are in a period of growth with heterogeneous sensitivity to drug therapy, ATs are generally drug-resistant and difficult to control with medical therapy alone. According to available studies (2, 3), while AT resolves spontaneously or can be easily controlled by medication in most children under 3 years of age, the spontaneous remission rate of those who have an onset after the age of 3 years is less than 20%, and they are more inclined to be drug refractory. In this study, 22 patients (78.6%) received standardized AAD therapy for at least 1 month, but existing AT remained in all patients before the procedure, supporting the view that ATs are often medically refractory. Thus, RFCA has irreplaceable advantages as a better treatment option for this group of children (6). In recent studies, three-dimensional mapping systems have further decreased fluoroscopy exposure during RFCA for AT substrates, resulting in higher success rates (approximately 80%–96%) and lower recurrence rates (0%–16%), as well as better identification and ablation of structurally-complex structures and isthmuses of AFL (12, 18–22).

### 4.2 RFCA treatment of AT

FAT accounts for the largest percentage of ATs in children. Unlike in adults, ectopic pacemaker cells in the atrial appendage that do not degenerate completely tend to be the origin of spontaneous FAT, other than the damaged atrial myocardium, in children (especially those aged <7 years) (23). The Pediatric Radiofrequency Catheter Ablation Registry of the Society of Pediatric Electrophysiology reported a lower RFCA success rate for treatment of FAT compared to that of other SVTs, whereas ablation of AATs had the lowest ablation success rate and the highest recurrence rate within 6 months after the procedure (24). Due to the complicated morphology, high mobility, relatively low blood flow, and thin wall, the duration of RFCA for AATs tends to be longer. These characteristics make RFCA for AATs more challenging for cardiac electrophysiologists (20). In our study, FAT originating in the atrial appendage accounted for the largest proportion (32%) of cases, and acute success was achieved in 24 of 25 patients (96.0%). During follow-ups, all recurrent cases appeared within 1–3 months after the procedure, and the difficulty of the ablation procedure for AAT was significantly higher than that of non-AAT cases (*p* = 0.01). These results are consistent with the conclusions of previous studies. Percutaneous pericardial ablation has been selected as an alternative treatment for cases of failure and recurrence following RFCA. Recently, atrial appendectomy has also been shown to be indicated in such cases. In a retrospective study (25), 71.4% (10/14) of AATs refractory to RFCA were eliminated after an atrial appendectomy. However, these treatments are more invasive and prone to postoperative complications; therefore, they are only recommended after poor RFCA outcomes.

AFL is a rare type of atrial arrhythmia in children, characterized by rapid and regular atrial activity (240–500 beats per minute) that occurs in the presence of atrial macroreentrant circuits within the atrial wall. As in adults, AFL in children is classified into typical (CTI-dependent) and atypical forms (26). AFL without congenital heart disease occurs predominantly in neonates and is uncommon in children or adolescents, except in those with congenital heart defects or a history of atrial surgery (27). According to previous studies, AFL is generally more difficult to control with drugs, but it is easier to achieve a permanent cure with ablation.

Owing to its high success rate and low risk of post-procedural complications, RFCA is recommended as the first-line therapy for children with AFL. Among these patients, the central obstacles of the circuit include CTI (mostly), scar tissues, suture lines, or patches, resulting in a slow conduction zone in the atrium (13, 26). In simple congenital heart defects, the circuit is formed either by the CTI or along the inferior lateral free wall of the right atrium; however, after cardiac surgery, free-wall incision scar-dependent AFL is more common. Moreover, improved outcomes can be achieved after ablating postoperative atrial incisional scars and the potential isthmus for scar-dependent AFL together (21). Four patients with AFL were included in this study, and all of them were found to have CTI-dependent AFL on electrophysiological examination, including one who had an AFL circuit formed around the right atrial surgical incision. One patient had a history of cardiac surgery, another had a history of fulminant myocarditis combined with an unclosed patent foramen ovale. A postoperative diagnosis of arrhythmogenic cardiomyopathy was made after RFCA as mentioned above. Their history was consistent with the opinion that most children with AFL have underlying cardiac pathologies (14).

RFCA is difficult to perform in children because of the narrow vessels. Similarly, procedures such as AAT ablation and perforation of the interatrial septum carry the risk of cardiac perforation and compression, and coronary artery manipulations may expose the coronary sinus to perforation or stenosis. The potential complications of RFCA for pediatric ATs are varied (12); however, they are relatively rare under strict and delicate procedural control, and range from 3% to 8% depending on the age of the patient. Common complications include 2° and/or 3° AV block, perforation, pericardial effusion, thrombosis, phrenic nerve injury, stroke, and cardiac arrest (1). In this retrospective study, no significant complications were observed. Acute success was achieved in 96.4% of the patients (27/28), and the overall success rate of RFCA was 89.3% (25/28) after long-term postoperative follow-up, which is similar to the rate reported in a large retrospective study conducted by Krause et al. (20), further confirming the safety and effectiveness of RFCA for treating pediatric AT.

### 4.3 Treatment and outcomes of TIC

TIC is a reversible cardiomyopathy caused by tachycardia and characterized by left ventricular remodeling and its dysfunction, which further increases the tachycardia burden and the difficulty of treatment (4, 5). According to previous studies, owing to its insidious onset and limited expression, children with underlying AT are more likely to develop TIC, placing them at higher risk of serious cardiac events and even sudden death (28). Approximately 28%–30% of children with ATs develop TIC weeks to years after the onset of AT, especially if it is incessant (29, 30). All patients with TIC in this study received pharmacological treatment at the time of diagnosis, and 75% (6/8) did not respond well to treatment. In contrast, improvements in cardiac function were seen within 1–2 days after ATs were eliminated by RFCA, and further recovery was observed during follow-up. These outcomes suggest that the cardiac function of children with TIC is reversible after the elimination of ectopic foci using RFCA. Although decreases were observed in the LVDd, there were no statistically significant differences before and after all treatments. Although dilation was not remarkable in these patients, this may suggest that the myocardial effects of TIC were profound and long-lasting. Recent studies have reported similar findings, including differences in cardiac structure and function among patients with TIC induced by ATs, with cardiomyocytes continuing to show systolic dysfunction and diffuse fibrosis after successful ablations (31, 32). In other words, diastolic dysfunction, ventricular dilatation, and histopathological abnormalities may persist despite the normalization of LVEF after RFCA. Therefore, early recognition of TIC is critical to avoid ventricular dysfunction and can reverse cardiac dysfunction with associated morbidity and healthcare burden, as well as improve the quality of life and long-term prognosis of patients. RFCA should be administered early as an effective permanent treatment (28).

## 5 Study limitations

This retrospective study was a single-center study with a limited number of included cases owing to the rare prevalence of AT. In addition, not all types of ATs were included in this study; therefore, multi-center studies with larger sample sizes and inclusion of different types of AT are required to determine whether RFCA is safe and effective for all types of ATs. Determining the appropriate timing of radiofrequency ablation therapy is a great challenge, especially for children who are drug-refractory and have coexisting TIC; therefore, a better understanding of the pathogenesis and pathophysiological changes associated with AT and TIC is essential.

## 6 Conclusion

This study demonstrates that RFCA is safe and effective for the treatment of different types of pediatric AT (including FAT and AFL), even during long-term follow-up. Patients who do not respond well to medication and have coexisting TIC should undergo RFCA earlier in the course of treatment. Future studies should focus on how to diagnose these patients early and treat them promptly.

## Data Availability

The original contributions presented in the study are included in the article/Supplementary Material, further inquiries can be directed to the corresponding author.
